# Evaluation of the Prognostic Value of Solute Carrier Family 34 Member 2 “SLC34A2” in Papillary Thyroid Carcinoma: An Immunohistochemical Study

**DOI:** 10.1155/2021/3198555

**Published:** 2021-07-14

**Authors:** Sarah Adel Hakim, Rasha Mohamed Abd El Atti, Reham Mohamed Faheim, Hoda Hassan Abou Gabal

**Affiliations:** ^1^Assistant Professors of Pathology, Faculty of Medicine, Ain Shams University, Egypt; ^2^Lecturer of Clinical Oncology, Faculty of Medicine, Ain Shams University, Egypt

## Abstract

**Background:**

Papillary thyroid carcinoma (PTC) usually has an indolent clinical course, yet a subset of patients might show an aggressive course. Thus, better stratification of at-risk patients is mandatory for proper management. Solute carrier family 34 member 2 (SLC34A2) is an independent prognostic indicator in several cancers. However, only a few studies have been conducted to evaluate the prognostic value of SLC34A2 in PTC, with none of them assessing its immunohistochemical (IHC) expression in a large cohort of patients with PTC or exploring its possible relationship with tumor progression. *Aim of the Study*. We aimed to evaluate the IHC expression of SLC34A2 in a large series of PTC patients, correlate its expression with established clinicopathological factors, and find any possible relationship between this marker and patient prognosis. *Material and Methods*. A total of 476 samples (including 238 samples of PTC and 238 samples of normal thyroid tissue) collected between 2002 and 2005 were extracted from the archives of the Pathology Lab, Ain Shams University Hospitals. IHC analysis was performed using an anti-SLC34A2 antibody. Follow-up data were obtained.

**Results:**

High SLC34A2 expression significantly correlated with important adverse clinicopathological parameters of PTC—i.e., late tumor stage, positive extrathyroid extension, tumor size  >  4 cm, and age  ≥  55 years (*p* ≤ 0.001 for each). Kaplan–Meier analysis revealed that high SLC34A2 expression significantly correlated with shorter disease-free survival (DFS; *p* = 0.005), but not with overall survival (*p* = 0.111). Multivariate analysis showed SLC34A2 to be an independent prognostic factor affecting DFS.

**Conclusions:**

High SLC34A2 IHC expression correlated with adverse clinicopathological prognostic parameters. Furthermore, SLC34A2 was identified as an independent factor for DFS that could serve to improve risk stratification of PTC patients for better management.

## 1. Introduction

Papillary thyroid carcinoma (PTC) is the most commonly encountered subtype of thyroid cancer, with increased incidence in recent decades [1–3]. Although it generally shows an indolent clinical course, there are patients with aggressive PTC at presentation who are most likely to develop local recurrences and distant metastases with unfavorable outcomes. Therefore, identifying this subset of patients becomes a top priority for the proper management of PTC, highlighting the importance of finding efficient prognostic biomarkers and new therapeutic targets in this context [4, 5].

Solute carrier family 34 member A2 (SLC34A2) is the most recognized member of solute carrier family 34. It is a sodium-dependent phosphate transporter that imports phosphate into cells, including tumor cells. Inorganic phosphate is crucial for different cell functions. SLC34A2 has a dual nature, acting as both a tumor suppressor and a tumor promoter in a context-dependent manner and therefore exhibiting upregulation in some tumors and downregulation in others [5–7].

Only a few studies have examined the SLC34A2 gene in PTC by real-time polymerase chain reaction or alternative techniques, demonstrating increased SLC34A2 gene expression in PTC [5, 8, 9]. No immunohistochemical (IHC) studies have been conducted on large cohorts to evaluate SLC34A2 expression in PTC. Moreover, none of the previous studies has assessed the possible role of SLC34A2 on tumor progression. Thus, the current study was aimed to evaluate the IHC expression of SLC34A2 in a large series of patients with PTC, correlate its expression with established clinicopathological prognostic parameters of PTC, and find any possible relationship between this marker and patient prognosis.

## 2. Material and Methods

### 2.1. Tissue and Patient Data

A total of 476 samples (including 238 samples of PTC and 238 samples of normal thyroid tissue) collected between 2002 and 2005 were extracted from the archives of the Pathology Lab, Ain Shams University Hospitals. Only cases with enough tissue were included in the analysis. Hematoxylin and eosin-stained slides were examined to evaluate and verify the histopathologic diagnosis. Follow-up data were obtained from the archives of Clinical Oncology Department to determine (a) overall survival time (OS), which was calculated from the date of diagnosis until the date of last follow-up or death, and (b) disease-free survival (DFS), which was calculated from the date of surgery to the date of progression (local recurrence or distant metastasis).

### 2.2. Ethics Statement

All patients who participated in this study signed written informed consent before surgery. The study was approved by the Research Ethical Committee at the Faculty of Medicine, Ain Shams University.

### 2.3. IHC Staining

Four-micrometer sections of formalin-fixed and paraffin-embedded samples of PTC and normal thyroid tissue were prepared for IHC staining with primary antibodies, e.g., rabbit monoclonal anti-SLC34A2 antibody (clone: SP322 N-terminal (ab228474); ABCAM, MA, USA; 1 : 100 dilution). Next, the avidin-biotin immunoperoxidase complex technique was employed as described by Hsu et al. [10] by means of a sensitive detection kit (Biogenex, CA, USA). The tissue sections were then subjected to a fixation on poly-L-lysine-coated slides overnight at 37°C, followed by deparaffinization and rehydration. After antigen retrieval in a microwave oven in 10 mM citrate buffer (pH 6.0) for 20 min, endogenous peroxidase activity was blocked using 3% hydrogen peroxide, and the sections were treated with Protein Block Serum-Free Solution (Dako Cytomation, Glostrup, Denmark) for 20 min and were subsequently incubated overnight at 4°C with primary antibodies. Afterward, biotinylated anti-mouse immunoglobulin and streptavidin conjugated to horseradish peroxidase were applied, and 3,3′-diaminobenzidine was used as a chromogen substrate to form an insoluble brown product. Finally, the sections were counterstained with hematoxylin and permanently mounted. With each run, sections of lung adenocarcinoma were used as positive controls for SLC34A2 [11]. Negative control sections were incubated with normal mouse serum instead of the primary antibody.

### 2.4. Interpretation of IHC Staining

Positive SLC34A2 IHC staining was detected in the membrane of tumor cells. SLC34A2 IHC expression was scored according to the percentage of positively stained tumor cells (0, <10%; 1, 10%–40%; 2, 40%–70%; and 3, >70%) and the intensity of staining (0, negative; 1, yellowish; 2, light brown; and 3, dark brown). A final immunoreactivity score was obtained for each case by multiplying the percentage of stained cells score by the intensity score (0–9) [5, 12, 13]. IHC analysis of SLC34A2 was blindly performed by 3 pathologists without any prior knowledge of the clinicopathological data. Any discrepancy between the 3 pathologists was resolved using a multihead microscope to reach a consensus.

Immunoreactivity for SLC34A2 was classified into 2 groups, namely, low expression (final score <  6) and high expression (final score ≥  6) [5].

## 3. Data Management and Analysis

Continuous variables were expressed as mean ± standard deviation, and categorical variables were reported as frequencies and percentages. Student's *t*-test was used to assess the statistical significance of differences between the 2 study groups. The chi-square test and Fisher's exact test were used to examine the relationship between categorical variables. OS and DFS were determined using Kaplan–Meier curves and compared by the log-rank test. We used a backward Cox regression model to compare time to specified events, taking into consideration the values of significant variables in univariate analysis. A significance level of *p* < 0.05 was used in all tests. All statistical procedures were carried out using SPSS version 20 for Windows (SPSS Inc., Chicago, IL, USA).

## 4. Results

A total of 238 cases of PTC were included in the current study, of whom 48 were males (20.2%) and 190 were females (79.8%). The mean age was 43.62 ± 12.77 years (range, 18–86 years). PTC variants were found to be classic (*n* = 172), follicular (*n* = 51), tall cell (*n* = 10), and solid (*n* = 5). Detailed clinicopathological characteristics are presented in Table 1.

Papillary thyroid carcinoma (PTC); standard deviation (SD).

### 4.1. IHC Analysis

**Figure 1:**
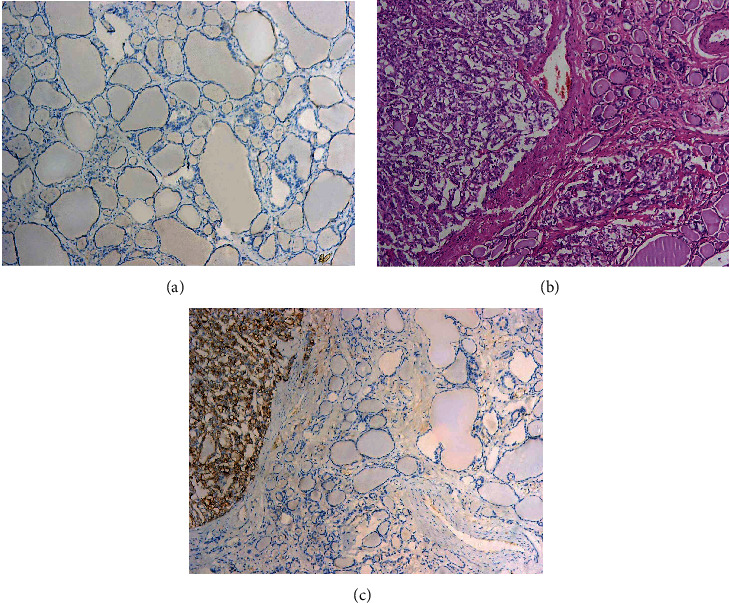
Normal thyroid tissue (control): showing low SLC34A2 IHC expression (IHCX100) (a), PTC with adjacent normal thyroid tissue: (H&EX100) (b), and PTC showing high SLC34A2 IHC expression in contrast with low SLC34A2 IHC expression in adjacent normal thyroid tissue (IHCX100) (c).

**Figure 2:**
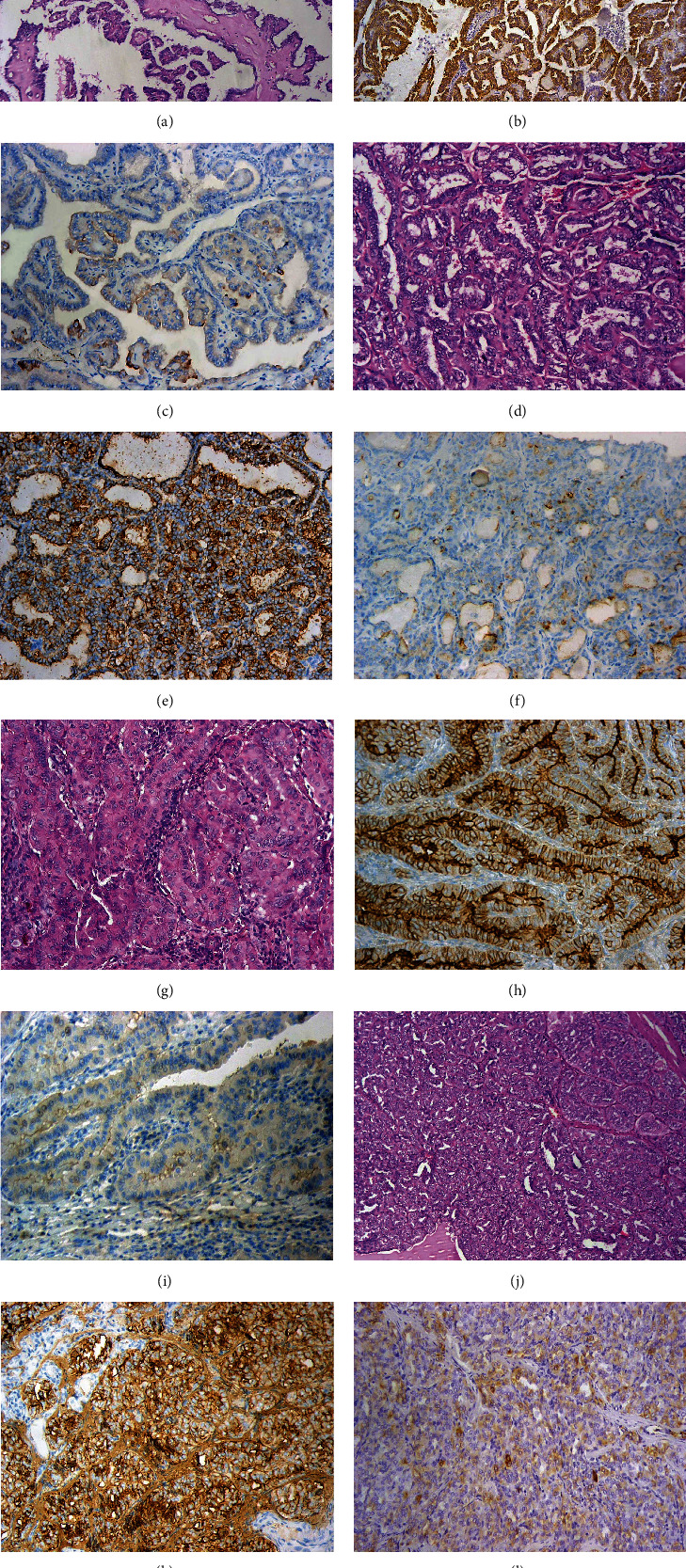
Papillary thyroid carcinoma (PTC) cases. Classic variant of PTC (H&EX100) (a). A case of classic PTC showing high SLC34A2 IHC expression (IHCX100) (b). Another case of classic PTC showing low SLC34A2 (IHCX200) (c). Follicular variant of PTC (H&EX200) (d). A case of follicular variant of PTC showing high SLC34A2 IHC expression (IHCX200) (e). Another case of follicular variant of PTC showing low SLC34A2 IHC expression (IHCX200) (f). Tall cell variant of PTC (H&EX200) (g). A case of tall cell variant of PTC showing high SLC34A2 IHC expression (IHCX200) (h). Another case of tall cell variant of PTC showing low SLC34A2 IHC expression (IHCX200) (i). Solid variant of PTC (H&EX100) (j). A case of solid variant of PTC showing high SLC34A2 IHC expression (IHCX200) (k). Another case of solid variant of PTC showing low SLC34A2 IHC expression (IHCX100) (l).

All control samples of normal thyroid tissue (*n* = 238), as well as normal thyroid tissue adjacent to the tumor, showed low SLC34A2 IHC expression (Figure 1). On the other hand, a significant elevation of SLC34A2 expression was detected in 68.5% of PTC cases, with high membranous SLC34A2 expression observed in 163 out of 238 PTC cases (Figure 2).

### 4.2. Correlation between SLC34A2 Expression and Clinicopathological Parameters

High SLC34A2 expression was found to be highly significantly associated with advanced tumor stage, positive capsular invasion, positive extrathyroid extension, large tumor size (>4 cm), and age ≥  55 years (*p* ≤ 0.001 for each). However, there was no statistically significant relationship between SLC34A2 IHC expression and PTC histopathological variants (*p* = 0.988), lymph node status at presentation (*p* = 0.484), and lymphovascular invasion (*p* = 0.595) (Table 2).

### 4.3. Survival Analysis

**Figure 3:**
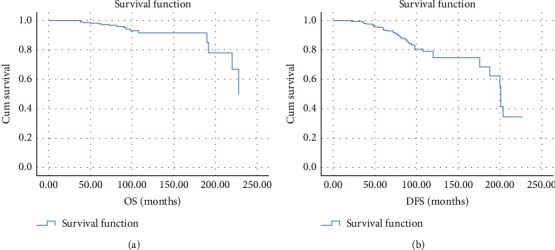
Kaplan Meier analysis of all PTC cases: overall survival (OS) (a) and disease-free survival (DFS) (b).

The OS of all PTC cases included in this study was 91.7% at both 10 and 15 years, while their 10- and 15-year DFS turned out to be 74.7% and 68.5%, respectively (Figure 3).

**Figure 4:**
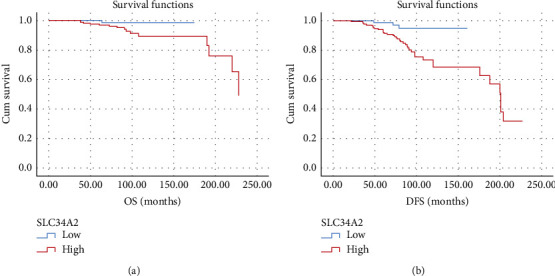
Kaplan Meier analysis of SLC34A2 IHC expression: correlation with OS is not significant (*p* = 0.111) (a) and high SLC34A2 correlates with shorter DFS (*p* = 0.005) (b).

OS at 175 months was 89.3% for PTC cases with high SLC34A2 expression vs. 98.6% for those with low SLC34A2 expression (*p* = 0.111), suggesting there was no statistically significant relationship between SLC34A2 IHC expression and OS. On the contrary, cases with high SLC34A2 IHC expression had significantly shorter DFS at 160 months than those with low SLC34A2 expression (68.4% vs. 94.8%, respectively; *p* = 0.005) (Figure 4).

After adjustment for significant factors affecting OS (i.e., age group, lymph node involvement at presentation, extrathyroid extension, tumor stage, tumor size, and SLC34A2 IHC expression) by backward Cox regression analysis, lymph node involvement at presentation and tumor stage were identified as independent factors affecting OS (Table 3). By contrast, SLC34A2 IHC expression was not found as an independent factor affecting OS.

After adjustment for significant factors affecting DFS (i.e., age group, lymph node involvement at presentation, capsular invasion, extrathyroid extension, tumor stage, tumor size, and SLC34A2 IHC expression) by backward Cox regression analysis, lymph node status at presentation, SLC34A2 IHC expression, and age were identified as independent factors affecting DFS (Table 4).

## 5. Discussion

Proper management of PTC requires better stratification of patients so that those with potentially aggressive outcomes can be promptly identified. Therefore, searching for new prognostic biomarkers and therapeutic modalities is essential for improving the prognosis of PTC. SLC34A2 is among the genes that have previously been linked to PTCs associated with the BRAF mutation. Previous studies have shown elevated SLC34A2 gene expression in PTC patients associated with BRAF^v600E^ mutations [8, 14]. Moreover, another study has even demonstrated that the SLC34A2 gene is downregulated in BRAF wild-type PTCs [15].

In the current study, a low level of SLC34A2 IHC expression was detected in all normal thyroid tissue samples, whereas there was a sharp rise in the IHC expression of SLC34A2 among PTC cases, with 68.5% of them displaying high SLC34A2 membranous expression. In this context, the only study investigating the IHC expression of SLC34A2 in PTC [5] found similar results, showing SLC34A2 IHC expression was significantly increased among PTC cases (64.4%) compared with normal thyroid tissue. The slight discrepancy in the percentage of expression might be attributed to their smaller sample size compared to ours. It is known that SLC34A2 might possess oncogenic or tumor-suppressive capabilities, therefore displaying different patterns of expression depending on cancer type [5]. In our study, the IHC expression of SLC34A2 increased in PTC samples as compared to normal thyroid tissue, which is similar to the expression pattern observed in ovarian cancer [16], breast cancer [17, 18], and osteosarcoma [19]. Nevertheless, SLC34A2 has been reported to be downregulated in some other cancer types, namely, renal cell carcinoma and nonsmall cell lung cancer [20, 21].

The present work revealed that higher SLC34A2 IHC expression was associated with poorer prognostic indicators of PTC, including older age, late tumor stage, larger tumor size, positive extrathyroidal extension, and positive capsular invasion (*p* ≤ 0.001 for each). In the same vein, several studies exploring SLC34A2 expression in other cancers have revealed a correlation between this marker and different clinicopathological parameters [18, 22]. In bladder cancer, for instance, SCL34A2 expression correlated with advanced tumor stage and large tumor size [23]. On the other hand, in the current work, high SLC34A2 IHC expression was not significantly associated with lymph node status at presentation and lymphovascular invasion (*p* = 0.484 and 0.595, respectively). These results are not in line with those reported by He et al. [5], who demonstrated that high SLC34A2 IHC expression was only associated with lymph node metastasis at presentation. This discrepancy might be attributed to the small number of PTC patients (*n* = 76) analyzed immunohistochemically in their study, where 36 out of 76 PTC cases (47.36%) presented with lymph node metastasis. It is worth noting that in our study, only 74 out of 238 PTC cases (31.1%) presented with lymph node metastasis, which is within the established international range of lymph node metastases at presentation in PTC cases (i.e., 30%–40% of PTC cases) [24]. Interestingly, SLC34A2 IHC expression did not correlate with PTC variants (*p* = 0.988), which might indicate a potential prognostic role for SLC34A2 in all PTC variants. This is unlike BRAF^v600E^, which showed less expression in the follicular variant of PTC [25]. This discrepancy between SLC34A2 and BRAF^v600E^ suggests that SLC34A2 could be linked to alternative molecular pathways that are yet to be explored.

Our results demonstrated that stage I tumors had a significantly lower level of SLC34A2 expression than did stage III tumors (61.9% vs. 100%, respectively; *p* ≤ 0.001). Similar significant differences in the expression of SLC34A2 existed between PTCs without extrathyroidal extension and those with extrathyroidal extension (64.3% vs. 96.8%, respectively; *p* ≤ 0.001), which might have resulted from the fact that SLC34A2 is presumably linked to PTCs associated with the BRAF mutation; hence, a possible explanation for the correlation between SLC34A2 and PTC invasiveness could be the ability of BRAF mutations to induce matrix remodeling genes [25].

In this study, SLC34A2 IHC expression was found to be correlated with gender (*p* = 0.013), with 123 out of 190 females exhibiting high SLC34A2 expression. The effect of gender on PTC prognosis has remained controversial. On the one hand, some studies have revealed that there is no difference between genders concerning disease-specific survival [26], and that male gender is not an independent prognostic factor for cancer-specific survival in PTC [27–29]; on the other hand, several more recent studies have demonstrated that male gender is an independent poor prognostic factor for PTC [30, 31]. Nonetheless, a subgroup analysis revealed that this might be altered by the effect of age and menopause-associated hormonal changes in older females [32], which could explain our current results since most of our cases were older females.

In the present study, Kaplan–Meier analysis demonstrated that high SLC34A2 expression did not significantly affect OS (*p* = 0.111). After adjustment for the other factors by backward stepwise Cox regression analysis, only lymph node status at presentation and tumor stage—but not SLC34A2 IHC expression—were identified as independent factors for OS. Yet, our results showed that patients with tumors expressing high levels of SLC34A2 had significantly shorter DFS (*p* = 0.005). Backward stepwise Cox regression analysis indicated that SLC34A2 IHC expression as well as age and lymph node status at presentation was independent factors affecting the DFS of PTC cases studied herein. This was in agreement with Han et al. [14] whose PTC cases that showed upregulated SLC34A2 gene expression had shorter relapse-free survival. Similarly, SLC34A2 expression has been reported as an independent factor for shorter DFS in CRC and bladder cancer [23]. A possible explanation for the significant effect of SLC34A2 on DFS, but not on OS, could be the fact that it is linked to BRAF mutations, which have been shown to significantly affect the DFS of PTC cases but not their OS. It could also be partly attributed to the fact that PTC patients generally have very long OS [33, 34].

The SLC34A2 gene product is the type II sodium-phosphate cotransporter (NaPi2b), which is highly expressed on the surface of PTC cells. Preclinical studies should be conducted to test the effectiveness of targeting this gene product by antibody–drug conjugates (ADCs) that can deliver cytotoxic agents to cells that express this specific cell marker (NaPi2b) [35]. Thus, anti-NaPi2b ADCs might be a promising therapeutic modality for the treatment of PTC. A recent study associating anti-NaPi2b antibody with BRAF mutations provided a theoretical basis for SLC34A2 inhibition and BRAF inhibition combined therapy which if validated and implemented might provide better management of PTC [14].

A limitation to this study is that the BRAF status of the included cases was not available. Hence, further studies should be conducted to evaluate the relationship between SLC34A2 and BRAF status in PTC.

In conclusion, to the best of our knowledge, this is the first IHC study to provide evidence that SLC34A2 expression is an independent factor facilitating PTC progression and metastasis. Moreover, it is the first IHC study conducted on a relatively large cohort of PTC patients that showed SLC34A2 expression was increased in PTC samples compared with control samples of normal thyroid tissue and was correlated with clinicopathological indicators of poor prognosis.

## 6. Conclusion

The overall data confirmed that high SLC34A2 IHC expression correlated with adverse clinicopathological prognostic parameters and could serve as an independent factor for DFS. Thus, it could be useful to improve the risk stratification of PTC patients to achieve better management.

## Figures and Tables

**(a):** 

	Mean	±SD (range)
Age	43.62	12.77 (18.00–86.00)
Tumor size (cm)	3.78	1.27 (1.00–7.00)

**(b):** 

		Number	%
Age group	<55	122	51.3%
	≥55	116	48.7%

Gender	Male	48	20.2%
	Female	190	79.8%

Lymph node involvement at presentation	N0	164	68.9%
	N1	74	31.1%

Lymphovascular invasion	Negative	192	80.7%
	Positive	46	19.3%

Capsular invasion	Negative	127	53.4%
	Positive	111	46.6%

PTC variants	Classic	172	72.3%
	Follicular	51	21.4%
	Other	15	6.3%

Extrathyroid extension	Negative	207	87.0%
	Positive	31	13.0%

Stage	I	189	79.4%
	II	38	16.0%
	III	11	4.6%

Tumor size (cm)	≤4	114	47.9%
	>4	124	52.1%

Distant metastasis	Negative	219	92.0%
	Positive	19	8.0%

Local recurrence	Negative	205	86.1%
	Positive	33	13.9%

Outcome	Dead	16	6.7%
	Alive	222	93.3%

**Table 2:** Relationship between SLC34A2 IHC expression and clinicopathological characteristics.

		SLC34A2				*p* value	Significance
		Low		High			
Age group	<55	56	45.9%	66	54.1%	≤0.001^∗^	HS
	≥55	19	16.4%	97	83.6%		

Gender	Male	8	16.7%	40	83.3%	0.013^∗^	S
	Female	67	35.3%	123	64.7%		

Lymph node at presentation	N0	54	32.9%	110	67.1%	0.484^‡^	NS
	N1	21	28.4%	53	71.6%		

Lymphovascular invasion	Negative	59	30.7%	133	69.3%	0.595^‡^	NS
	Positive	16	34.8%	30	65.2%		

Capsular invasion	Negative	69	54.3%	58	45.7%	≤0.001^∗^	HS
	Positive	6	5.4%	105	94.6%		

PTC variants	Classic	54	31.4%	118	68.6%	0.988^∗^	NS
	Follicular	16	31.4%	35	68.6%		
	Other	5	33.3%	10	66.7%		

Extrathyroid extension	Negative	74	35.7%	133	64.3%	≤0.001^∗^	HS
	Positive	1	3.2%	30	96.8%		

Stage	I	72	38.1%	117	61.9%	≤0.001^∗^	HS
	II	3	7.9%	35	92.1%		
	III	0	0.0%	11	100.0%		

Tumor size (cm)	≤4	62	54.4%	52	45.6%	≤0.001^∗^	HS
	>4	13	10.5%	111	89.5%		

^‡^Student's *t*-test. ^∗^Chi-square test. Solute carrier family 34 member A2 (SLC34A2); immunohistochemical (IHC); papillary thyroid carcinoma (PTC); highly significant (HS); significant (S); not significant (NS).

**Table 3:** Backward Cox regression analysis of important factors affecting OS.

	Hazard ratio (HR)	*p* value	Significance	95.0% confidence interval for HR	
				Lower	Upper
Lymph node at presentation	8.623	≤0.001	HS	2.355	31.570
Tumor stage II/III^∗^	4.398	≤0.001	HS	2.220	8.711

^∗^Reference Tumor Stage I. Overall Survival (OS); Highly Significant (HS).

**Table 4:** Backward Cox regression analysis of important factors affecting DFS.

	Hazard ratio (HR)	*p* value	Significance	95.0% CI for HR	
				Lower	Upper
LN at presentation	10.404	≤0.001	HS	5.023	21.549
High SLC34A2^∗^	3.961	0.025	S	1.186	13.226
Age group ≥55^∗∗^	2.775	0.017	S	1.196	6.436

^∗^Reference low SLC34A2. ^∗∗^Reference age group < 55 years. Disease-free survival (DFS); lymph node (LN); confidence interval (CI); highly significant (HS); significant (S).

## Data Availability

All data generated or analyzed during this study is included in this published article.
